# From Glacier to Sauna: RNA-Seq of the Human Pathogen Black Fungus *Exophiala dermatitidis* under Varying Temperature Conditions Exhibits Common and Novel Fungal Response

**DOI:** 10.1371/journal.pone.0127103

**Published:** 2015-06-10

**Authors:** Barbara Blasi, Hakim Tafer, Donatella Tesei, Katja Sterflinger

**Affiliations:** VIBT-Extremophile Center, Department of Biotechnology, University of Natural Resources and Life Sciences, Vienna, Austria; Ruhr-University Bochum, GERMANY

## Abstract

*Exophiala dermatitidis (Wangiella dermatitidis*) belongs to the group of the so-called black yeasts. Thanks in part to its thick and strongly melanized cell walls, *E*. *dermatitidis* is extremely tolerant to various kinds of stress, including extreme pH, temperature and desiccation. *E*. *dermatitidis* is also the agent responsible for various severe illnesses in humans, such as pneumonia and keratitis, and might lead to fatal brain infections. Due to its association with the human environment, its poly-extremophilic lifestyle and its pathogenicity in humans, *E*. *dermatitidis* has become an important model organism. In this study we present the functional analysis of the transcriptional response of the fungus at 1°C and 45°C, in comparison with that at 37°C, for two different exposition times, i.e. 1 hour and 1 week. At 1°C, *E*. *dermatitidis* uses a large repertoire of tools to acclimatize, such as lipid membrane fluidization, trehalose production or cytoskeleton rearrangement, which allows the fungus to remain metabolically active. At 45°C, the fungus drifts into a replicative state and increases the activity of the Golgi apparatus. As a novel finding, our study provides evidence that, apart from the protein coding genes, non-coding RNAs, circular RNAs as well as fusion-transcripts are differentially regulated and that the function of the fusion-transcripts can be related to the corresponding temperature condition. This work establishes that *E*. *dermatitidis* adapts to its environment by modulating coding and non-coding gene transcription levels and through the regulation of chimeric and circular RNAs.

## Introduction

Black yeasts are a polyphyletic morphological group within the Ascomycetes that is characterized by melanized cells and yeast-like growth states (multilateral and polar budding cells) in addition to hyphal growth. Some species, like *E*. *dermatitidis*, exhibit meristematic growth and form morula-like colonies in animal and human tissue or in natural stone [1,2]. All black yeasts and meristematic fungi share a number of universally present characters, such as strong melanization, thick and even multi-layered cell walls and exo-polysaccharide production, resulting in an extraordinary ability to tolerate chemical and physical stresses such as extreme pH, high and low temperature and desiccation [3–5]. Moreover, some of those fungi—including *E*. *dermatitidis*—show increased cell growth—division and cell size—when exposed to ionizing radiation [6,7].

The black fungi associated with humans are represented by the typical black yeasts belonging to the genera *Exophiala*, *Fonsecaea*, *Capronia*, *Phaeococcomyces* and *Cladophialophora*. Phylogenetically these genera are accumulated in the order of Chaetothyriales and the family of Herpotrichiellaceae. Within this family the genus *Exophiala* seems to be an evolutionary hot spot with a high diversification and emerging adaptation towards animals—e.g. *Exophiala salmonis*—towards the human host, in the case of *Exophiala dermatitidis* and—or to human environments such as bathrooms, sauna facilities or dishwashers [8,9]. *E*. *dermatitidis* is of special medical importance since it causes a variety of severe illnesses in humans: it is a causative agent of keratitis, of subcutaneous phaeohyphomycosis, and of chromoblastomycosis, and may cause pneumonia. Further, the fungus is neurotropic and causes fatal brain infections, and it plays an important role in patients with cystic fibrosis [8,10–13].

For many years the black yeasts and the microcolonial fungi were regarded as typical inhabitants of extreme environmental habitats such as the phylloplane, or rock in semi-arid and desert environments [5,14–16]. However, species of *Exophiala* show high prevalence in the human environment and seem to be rather frequent in sauna and steam bath facilities, in sink drains, and in drinking water [17–19]. Examinations of bathwater and sludge in drainpipes that are warmed daily to over 42°C have identified several species of this medically important genus [20]. Recently, mass growth of dark fungal biofilms on water taps and associated habitats was observed in various German drinking water distribution systems [21,22]. Here, *E*. *lecanii-corni* was found to be the major component in 10 out of 13 biofilms analyzed. Biofilm-forming *Exophiala mesophila* has been isolated from chlorine-dioxide-treated dental unit waterlines. *E*. *dermatitidis* and *E*. *phaeomuriformis* were reported to form stable communities in dishwashers, and extensive global studies showed that more than 50% of all rubber or silicone sealings in dishwashers are colonized with these fungi [23,24]. This finding generated considerable public attention and raised many questions concerning the routes of entry, the natural reservoir and the virulence for the human host of this fungus.

Interestingly, besides the human related habitats which are characterized by rather high temperatures, strains of *E*. *dermatitidis* have been isolated from glaciers—e.g. the Calderone glacier in the Apennines [25]—as well as in the Arctic and Antarctic environments [26]. Furthermore, Gunde-Cimerman (personal communication) is suggesting that natural spring water is a possible source of the fungi and is the vehicle by which it enters the human environment. This ability to adapt to the temperatures and nutrients found in environments as diverse as cold glaciers, hot saunas and dishwashers, as well as the more temperate human body, requires an important phenotypic plasticity, that might, in turn, explain the success of *E*. *dermatitidis* as a human pathogen.

*E*. *dermatitidis*, due to its association to the human environment and due to its phylogenetic relation to many environmental black fungi (which are known as poly-extremophilic or poly-extremotolerant) has become an important model organism for system biology studies, including proteomics and transcriptomics [9,27]. However, the knowledge of the system biology of this fungus is still limited. In order to better understand the capacity of this fungus to adapt, we have reported the results of a transcriptomic study of gene expression responses under different temperature regimes and exposition times.

With this aim, *E*. *dermatitidis* was incubated at 1°C—thus simulating the cold environmental habitat—at 37°C—which is the optimal growth temperature for this strain—and at 45°C in order to simulate raised temperatures in the human environment—e.g. hot tap water.

## Materials and Methods

### Fungal strain and experimental conditions

*Exophiala dermatitidis* (CBS 525.76) was cultured in Malt extract agarose media (2% malt extract, 2% D-glucose, 0.1% bacto-peptone and 2% agar). For the RNA-seq experiment, the cells were grown in Petri dishes for 2 weeks at 37°C and then as follows: one week at 37°C (control, 37C), 1 week at 45°C (45C1W), 1 week at 37°C plus 1 hour at 1°C (1C1H) or at 45°C (45C1H), 1 week at 1°C (1C1W) and 1 week at 45°C (45C1W), for a total of four treatments.

### RNA-seq

Total RNA was extracted with FastRNA PRO RED KIT (MP Biomedicals, Santa Ana, CA) according to the instructions of the manufacturer. The mRNA was isolated with the Dynabeads mRNA DIRECT Micro Kit (Ambion by Life Technologies, Carlsbad, CA) and the following transcriptome library preparation was performed with the Ion Total RNA-Seq Kit v2 (Life Technologies, Carlsbad, CA).

Total RNA, isolated mRNA and the final cDNA library were all qualitatively and quantitatively evaluated by mean of Agilent 2100 Bioanalyzer (Agilent Technologies, Santa Clara, CA). The RNA-seq was realized by means of Ion Torrent technology coupled with the Ion Proton sequencer (Life Technologies, Carlsbad, CA). The average read length was 175 bp for all five samples. Total reads generated per sample varied between 57,611,573 and 99,965,344.

### Gene Annotations

Protein annotation and functional annotation were retrieved from previous publications [4,5,27]. Interproscan5 with default parameters was used to improve the Gene Ontology annotations [28].

NcRNAs were annotated based on methods using sequence and structure homologies. Infernal [29] with default parameters was used to find ncRNAs in a general way. tRNAs were annotated with tRNAscan [30] with default parameters. RNAmmer was used to find rRNAs genes [31] while snoBoard [32] was used to search for snoRNAs. Finally BLAST [33] was used to look for putative miRNAs, rRNAs, sRNAs. RnasP and RnaseMP. Overlapping non-coding loci from different families were kept. In cases where more than one member of the same ncRNA family (snoRNA, miRNA, snRNA, tRNA) were assigned to the same locus, the member with the highest similarity to the search model/search sequence was kept.

### Read Mapping and Analysis

Trimmed reads were downloaded from our local ion proton server. Segemehl [34] version 1.9 with the split-read detection option set, was used to map the reads on the recently published *E*. *dermatitidis* genome [27] while lack [34] was used to remap reads that were not mapped during the first mapping step. Lack utilizes *de novo* splice junction information from alignments reported by state-of-the-art split-read aligners. In contrast to other methods, lack is able to map reads across multiple splice junctions. Splice sites detection and classification was done with testrealign [35] with default settings. Reads that were split-mapped were assigned to one of the following three categories: “normal,” same strand, same chromosome and insert between 15 and 200 kb and matched fragments co-linear with the genomic DNA; “circular,” same strand, same chromosome and junction distance less than 200 kb with fragment order inverted relative to genomic DNA; “(strand)switched,” same chromosome, junction distance less than 200 kb and fragments located on opposite strands. Overlaps between mapped reads and annotation data were computed with the aid of BEDTools [36].

Fused transcripts were detected by looking at split reads connecting two different transcripts. In order to get high confidence fused transcripts we concentrated on split-reads whose ends were mapping less than 20nts from a canonical splice site, as suggested in [35]

. The presence of monoexonic circular transcripts, where a single intron or exon is circularized, was investigated by looking at split reads with the start and end located on the same exon/intron and with ends separated by less than 400nts. Furthermore, at least 8 reads should support the circular RNA and at least 50% of the spliced reads mapping at the splice has to support the circRNA.

Cuffdiff [37] was used to look for differential expression of genes, in a similar manner to the methods presented in [38]. The mapping output of segemehl was modified to fit the input requirements of cufflinks using a custom script. CummeRbund [39], a R module [40], was used to search for significant (at least 8 folds) differential expression between 37C and the other 4 temperature conditions (1C1H, 1C1W, 45C1H, 45C1W). AnnotationForge, GOstats [41], GSEABase [42] and KOBAS [43] were used to analyse GO and KEGG pathways enrichment of the up- and down-regulated genes.

Enriched GO terms and KEGG pathways with an uncorrected p-value < 0.05 were considered significant. REVIGO with default parameters was used to summarize the list of returned GO terms [44].

The mapping of the regulated genes on the pathways was performed with custom scripts using the KEGG database [45].

## Results

To understand the molecular mechanisms behind the acclimatization of *E*. *dermatitidis* under a wide range of temperature adaptations, the fungus was exposed to 45°C and 1°C and the gene expression was analyzed through RNA-seq technology. In order to investigate the fungal response to short (stress) and long-term changes (acclimatization), *E*. *dermatitidis* was exposed for one hour and for one week, respectively, at both temperatures. The control condition is represented by the exposure to 37°C.

Tests for thermal preferences conducted prior to setting up the experimental conditions, indicated 37°C as the optimum temperature for *E*. *dermatitidis* and showed the presence of fungal growth at both 45°C—as also previously demonstrated [23]—and at 1°C. In addition, the fungal colonies proved to be viable after prolonged treatment at all the selected temperatures (data not shown, Tesei et al. in preparation).

The transcriptome analysis was performed comparing pairs of experimental conditions, providing a list of over-represented Gene Ontology and KEGG terms of the differentially expressed genes. In order to deepen our knowledge of the dependence of cell regulation upon temperature changes, the annotation of *E*. *dermatitidis* was extended with ncRNA annotation. Furthermore, circular RNAs and trans-spliced genes were also studied.

### Overview of RNA sequencing

**Fig 1:**
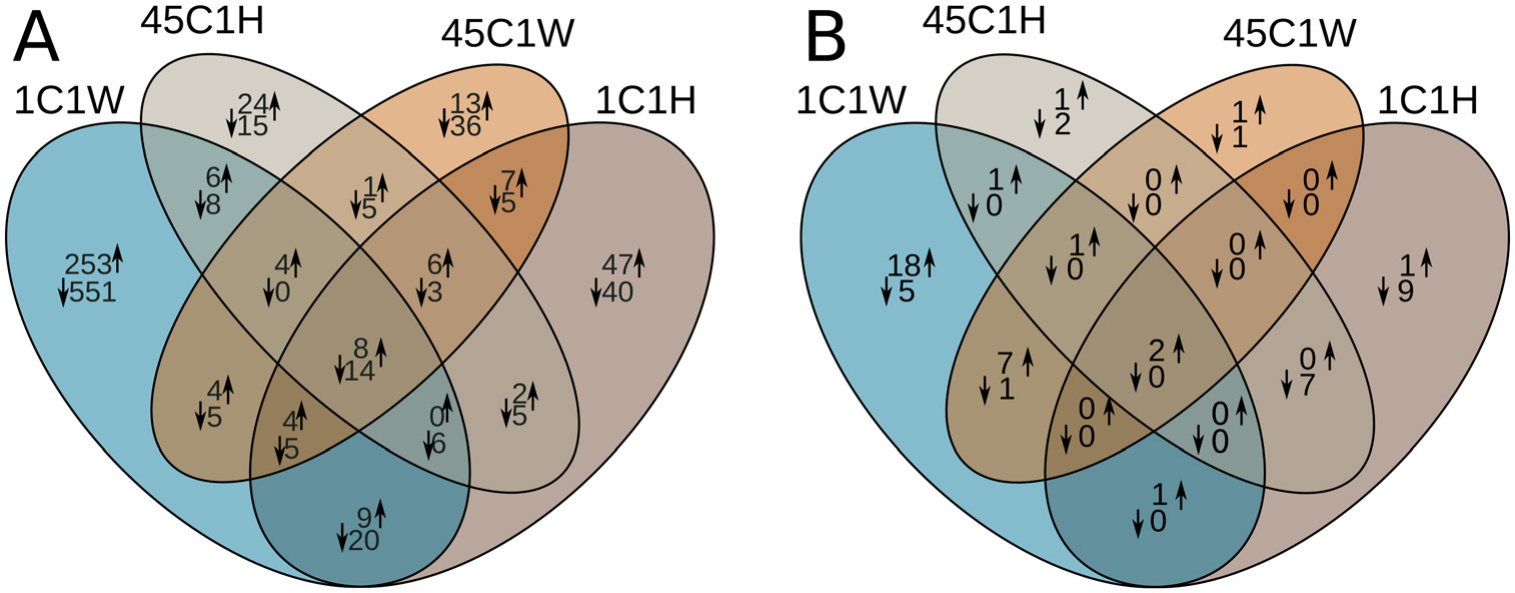
Representation of up- and down-regulated genes for cold and hot conditions, compared to 37°C. Upwards and downwards arrows represent up- and down-regulated genes, respectively. Protein coding genes (A) show the highest number of differentially expressed genes following the treatment at 1°C for 1 week (1C1W), with a total of 609 and 288 down- and up-regulated genes, respectively. NcRNA genes (B) show the highest number of differentially expressed genes at the same experimental condition (1°C for 1 week, 1C1W), with a total of 30 and 6 up- and downregulated genes, respectively. For both coding and non-coding genes, the smallest number of differentially expressed genes is found when the fungus have been exposed at 45°C for 1 week (45C1W).

**Fig 2:**
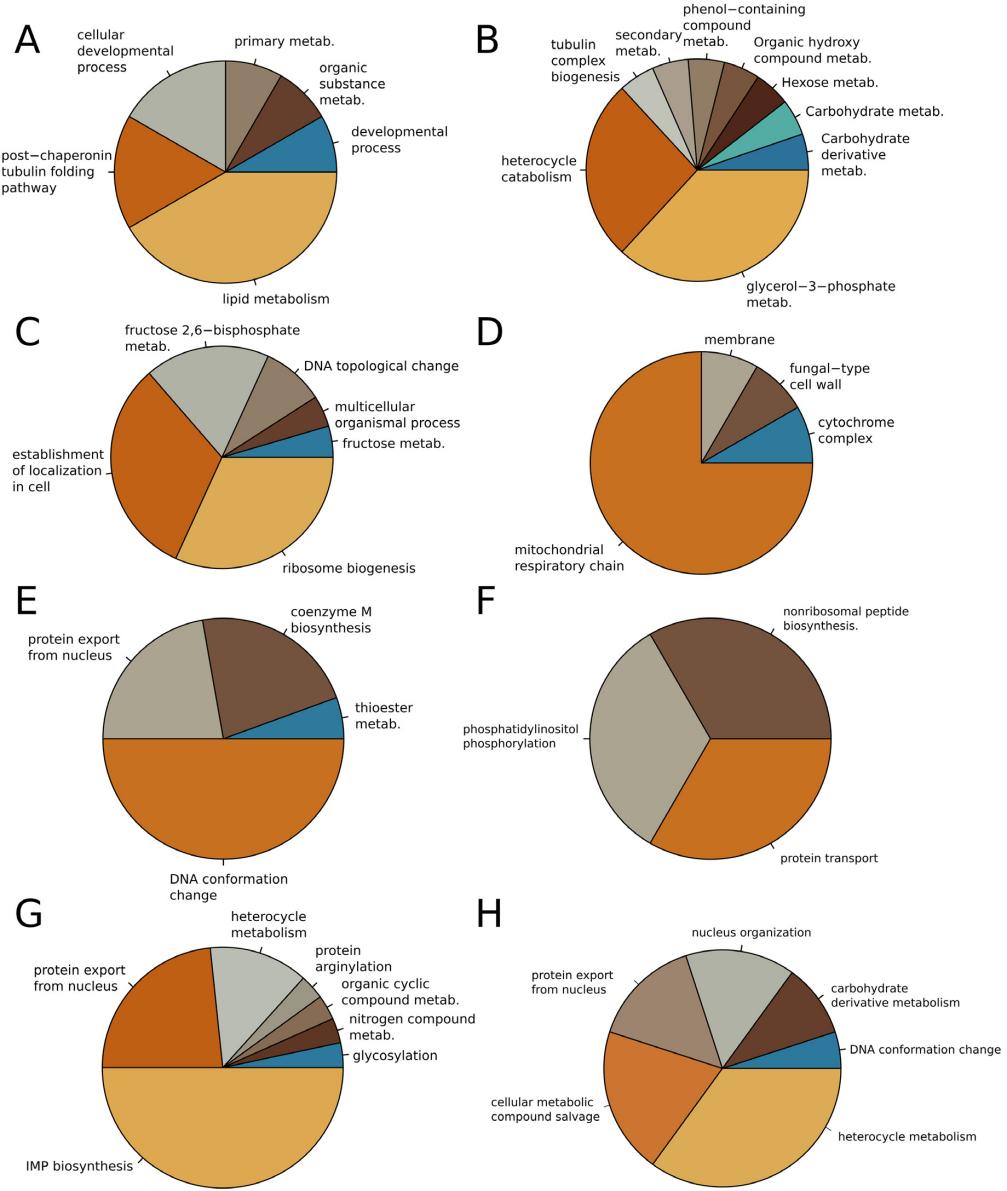
Pie chart of the summarized Gene Ontology (GO) terms for different conditions. (A) Over-represented BP-related GO terms for the genes upregulated at 1°C for 1 hour. (B) Over-represented GO terms related to biological processes (BP) for the genes up-regulated at 1°C for 1 week. (C) Over-represented BP-related GO terms for the genes down-regulated at 1°C for 1 week. (D) Over-represented CC-related GO terms for the genes up-regulated at 1°C for 1 week. (E) Over-represented BP-related GO terms for the genes down-regulated at 45°C for 1hour. (F) Over-represented BP-related GO terms for the genes up-regulated at 45°C for 1 week. (G) Over-represented BP-related GO terms for the genes down-regulated at 45°C for 1 week. (H) Over-represented BP-related GO terms for the genes up-regulated at both 45C1H and 45C1W.

In order to get an overview of the alteration of *E*. *dermatitidis* transcriptome upon temperature changes, the number of strongly (8 fold) up- and down-regulated coding and non-coding genes were derived by comparing their transcription level at 37C and 1C1W, 1C1H, 45C1H and 45C1W. While we are confident that our mRNA isolation protocol successfully selected polyA-transcripts, we also found reads coming from ncRNAs loci. This is in line with previously- reported polyadenilated ncRNAs found in various prokaryotes and eukaryotes [46]. For the protein-coding genes, the largest number of differentially expressed genes is found for 1C1W, where 609 genes were down-regulated and 208 genes were up-regulated. The smallest number of differentially expressed genes is found for 45C1W, where only 72 and 45 genes were down- and upregulated, respectively (Fig 1A). Pie charts with up- and downregulated GO terms were produced for each temperature exposure in comparison with the control. The most informative are represented in Fig 2 and the others are listed in the supporting information.

Similar to the protein-coding genes, the highest number of differentially expressed ncRNAs is found for 1C1W, where in contrast to proteins, more genes were up-regulated (30) than down-regulated (6). As for the coding genes, the smallest number of strongly regulated genes is found for 45C1W (Fig 1B).

### Exposure to 1°C compared to 37°C

After a short-term exposure to 1°C, the major changes in the metabolic profile of. *E*. *dermatitidis* are visible in the lipid metabolism, and in particular the glycero- and glycerophospho-lipids (Fig 2A and S1 Table). Post-chaperonin tubulin folding pathway and cellular development are the next up-regulated processes in this dataset (Fig 2A and S1 Table). This is in line with results showing that cold shock response induces genes related to cytoskeleton rearrangement [47,48]. Genes related to the cell cycle and DNA-checkpoints are also up-regulated (S1 and S10 Tables).

In contrast, processes related to the nuclear envelope, the protein export from the nucleus, the protein-DNA complex and the DNA conformation change are down-regulated (S2 Table). Moreover, *E*. *dermatitidis* reacts to 1°C temperature by slowing down mitosis and the consequent cellular division, as well as the transcription machinery.

After exposure to 1°C for 1 week the metabolism of glycerol 3-phosphate (G3P) was strongly upregulated (Fig 2B and S3 Table). Other overrepresented BP terms of upregulated genes are related to hexose, carbohydrate and carbohydrate derivative metabolism (S3 Table).

Similarly to the 1C1H stress condition, *E*. *dermatitidis* down-regulates the ribosome biogenesis and rRNA processing at 1C1W (Fig 2C), indicating that, in general, cold stress represents a stimulus for slowing down the transcription machinery. We also see a concomitant decrease of histone acetylation (p = 0.035), known to be correlated with decreased levels of gene expression [49]. The nuclear transport and nuclear organization are highly represented among the down-regulated genes, confirming that the fungus had slowed down nuclear activity. Exocytosis, membrane docking and vesicle mediated transport are also down-regulated processes. Finally, on the cellular component level, there is an enrichment of terms related to the cellular and mitochondrial respiratory chain for the up-regulated genes (Fig 2D), while for the down-regulated genes, the nuclear envelope is over-represented (S11 Table).

We examined further the genes that were similarly regulated during the short and long exposure to 1°C compared to 37°C. At 1°C, the genes that are commonly up-regulated are related to the lipid metabolism, post-chaperonin tubulin folding pathway and cellular developmental process. The common down-regulated genes are instead related to protein export from nucleus and purine nucleoside salvage.

### Exposure to 45°C compared to 37°C

After 1 hour exposure to 45°C, the up-regulated genes are all related to DNA metabolism. In particular, as shown in S5 Table, we could observe an over-representation of biological processes related to deoxyribose phosphate metabolism, alanyl-tRNA aminoacylation and DNA replication initiation, strongly indicating that the fungus is actively synthesizing DNA. This is further supported by the finding that the MCM (mini chromosome maintenance) complex, the core of eukaryotic replicative helicase, is also up-regulated (S14 Table). Among the down-regulated processes (Fig 2E and S6 Table) DNA conformational change was found, as well as DNA packing and mitotic chromosome condensation, indicating that short exposure to 45°C shifted the fungus into an active replication stage.

The main biological processes activated after a 1 week exposure to 45°C are related to phosphatidylinositol (PI) phosphorylation, protein transport and nonribosomal peptide biosynthesis (Fig 2F and S7 Table). Phosphatidylinositol phosphorylation leads to phosphatidylinositol 4-phosphate (PI-4-P) and the latter is known to be prevalent in Golgi apparatus membranes, where it is responsible for recruiting proteins that need to be carried to the cell membrane [50]. The large number of biological processes that are down-regulated at 45C1W, like the nuclear envelope organization or protein export from the nucleus, (Fig 2G and S8 Table for the complete list) seems to indicate a cell in a kind of quiescent state compared both to the exposure to its optimal temperature and to the stress exposure at 45°C.

No gene was found to be significantly down-regulated at both 45C1W and 45C1H compared to 37°C. On the other hand, 30 GO terms were found to be significantly over-represented in the group of genes that are strongly up-regulated at both 45C1H and 45C1W (Fig 2H). Those terms are related to DNA conformation change, protein export from the nucleus, cellular metabolic compound salvage, carbohydrate derivative metabolism; heterocyclic metabolism and nucleus organization.

Aside from comparing two temperature conditions, we also looked at genes with similar expression patterns along the temperature profile with cummeRbund [51]. We concentrated on the group of genes with high expression at low temperature and low expression at 45°C (S3 Fig). Interestingly the genes in this cluster were enriched in GO terms related to trehalose, a potent cryoprotectant whose concentration was shown to increase with decreasing temperature [47,52]. HMPREF1120_00310, one of the member of the cluster, is involved in the desaturation of membrane fatty acid, a process that fluidizes the cell membrane at low temperature [53,54].

### Pathway analysis

Similar to the GO enrichment terms, KEGG pathway enrichment was performed for all four conditions. In contrast to the GO terms analysis, significant enrichment of KEGG terms was only found for genes down-regulated at 1C1W. Under this condition the fatty acid degradation pathway, as well as the tryptophan metabolism, contain significantly more down-regulated genes than expected, with p-values of 3.3x10^-6^ and 1.6x10^-7^, respectively. While fatty acids are of utmost importance for the regulation of the cell membrane fluidity, it was only recently shown that tryptophan plays a role in fungi under cold conditions [55].

Pathways related to cell wall biosynthesis, cell wall integrity, melanin biogenesis and trehalose metabolism, were analyzed for genes that were highly (more than eight-folds) up- or down-regulated.

The highest number of pathways containing genes strongly regulated was seen again at 1C1W. For example, under that condition, the three melanin pathways contain mainly down-regulated genes (S17 Table) indicating that melanin production is probably diminished under cold condition.

From the 6 pathways related to cell wall stress response, only 1,3-α-glucan synthesis and processing and 1,3-β-glucan synthesis and processing showed important regulation of their genes upon temperature shift. More precisely Alpha-amylase (HMPREF1120_03460), the only significantly regulated gene in the 1,3-α-glucan synthesis and processing pathway, was up-regulated at 1C1W, in line with results previously found in wheat grain [49].

From the 4 differentially expressed genes in the 1,3-β-glucan synthesis pathway, Glucan 1,3-beta-glucosidase, extracellular cell wall glucanase Crf1 and Glycosyl transferase are down-regulated at 1C1W, while endo-1,3(4)-beta-glucanase is up-regulated at 1C1W.

Trehalose is very well known for its involvement in abiotic stress resistance in plants and fungi and its synthesis has been related to salt, drought, heat and cold stress in many organisms [47,52,56]. Since the induction of trehalose synthesizing enzymes is a typical response to near freezing temperatures in yeasts [47,52], we pointed out which genes of the trehalose metabolism were regulated in the case of long-term exposure to 1°C. Trehalose is synthesized through the processing of α-D-glucose-1P. The pathway starts with α-D-glucose-1P which is converted by UTP-glucose-1-phosphate transferase, an enzyme that is expressed four times more at 1C1W than at 37C, to UDP-glucose. UDP-glucose is then processed by UDP-glucose 6-dehydrogenase or glycogen synthase, two enzymes that do not lead to trehalose, or by trehalose 6-phosphate synthase, leading to trehalose-6P and subsequently to trehalose. At 1C1W the 6-dehydrogenase and glycogen synthase enzymes are down-regulated by a factor of at least 4, increasing the amount of UDP-glucose to be processed by trehalose 6-phosphate synthase, which is itself up-regulated by a factor of at least 4, into trehalose-6P and subsequently into trehalose (S1 Fig).

### Fused transcripts

**Fig 3:**
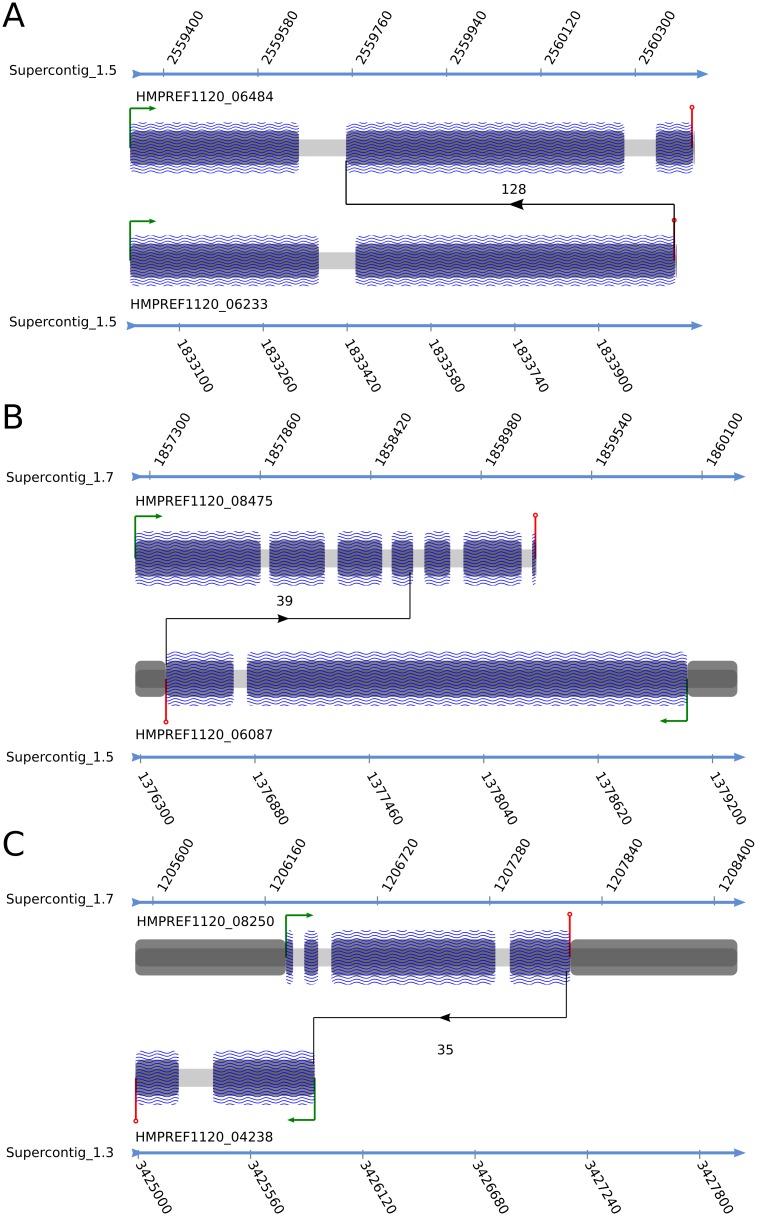
Graphical representation of the chimeric RNAs with the highest number of split reads for different temperatures. Fusion transcripts present at the following experimental conditions: 1°C for 1 week (A), 37°C (B) and 45°C for 1 week (C). The thick blue arrow represents the Supercontig with the corresponding scale. The start codon is represented by the green arrow. The stop codon is shown as a red vertical line. The splice connecting both genes is shown in black and the number of supporting split reads is located between both fused genes. The blue regions represent the Coding DNA Sequence (CDS), the dark grey regions represent the Untranslated Regions (UTRs), while the light gray regions represent introns. The splicing direction is given by the black arrow head.

A totally novel finding in *E*. *dermatitidis* is the presence of fused transcripts, i.e. annotated transcripts connected by split-reads. They are found under all conditions, with the highest and lowest number of fused transcripts found at 1C1W (873) and 37C (82), respectively (S19 Table). The number of split-reads and the corresponding connected genes were dependent on the experimental conditions. At 1C1W, the fused transcript with the highest number of split-reads (130) is made out of MC family mitochondrial carrier protein (HMPREF1120_06233) and L-fuculose-phosphate aldolase (HMPREF1120_06484) (Fig 3A). Aldolase was previously shown to be up-regulated under cold stress in *Arabidopsis thaliana* [57].

At 1C1H, the fusion transcript with the highest number of trans-splicing events (128) is composed of an amidase and translation initiation factor eIF-4F. At 37C, HMPREF1120_08475, an ankyrin-containing protein, is connected to V-type proton ATPase subunit A (HMPREF1120_06233), two proteins involved in the energy metabolism (Fig 3B). Interestingly, in the bat *Myotis brandtii*, V-type proton ATPase subunit B is reported to contain 3 ankyrin domains [58].

At 45C1H, the second coding exon of Glucose-6-phosphate isomerase (HMPREF1120_08503) is connected to the first coding exon of HMPREF1120_03038 through 36 split reads, an homolog to Adhesin protein Mad1. Finally at 45C1W, the whole coding region of HSP40 (HMPREF1120_08250), a molecular chaperone involved in the heat shock response, is ligated through 35 reads to the whole transcript of HMPREF1120_04238, a homolog of an uncharacterized Glycosylphosphatidylinositol (GPI)-anchored protein found in various fungi (Fig 3C). This fusion transcript, with its heat shock domain, fits nicely with the condition and with the up-regulated phosphatidylinositol phosphorylation process at 45C1W.

### NcRNAs annotation

A total of 221 ncRNAs were annotated in the genome. All major ncRNA families, with the exception of miRNAs, were found (S20 Table). Similar to the protein coding genes, ncRNAs are differentially expressed. The highest and lowest number of regulated genes are found at 1C1W (30 up- / 6 down-regulated) and 45C1W (11 up- / 2 down-regulated) (Fig 1B), similar to what was found for the protein coding genes. However, in contrast to mRNAs, at 1C1W more ncRNAs are positively regulated than negatively. Eight ncRNAs, all belonging to the class of snoRNA, are located in introns of protein coding genes. Previous reports on intronic snoRNAs [59] showed that the regulation pattern between the intronic snoRNAs and their host genes might differ, something that is also seen in *Exophiala dermatitidis* (S2 Fig).

**Fig 4:**
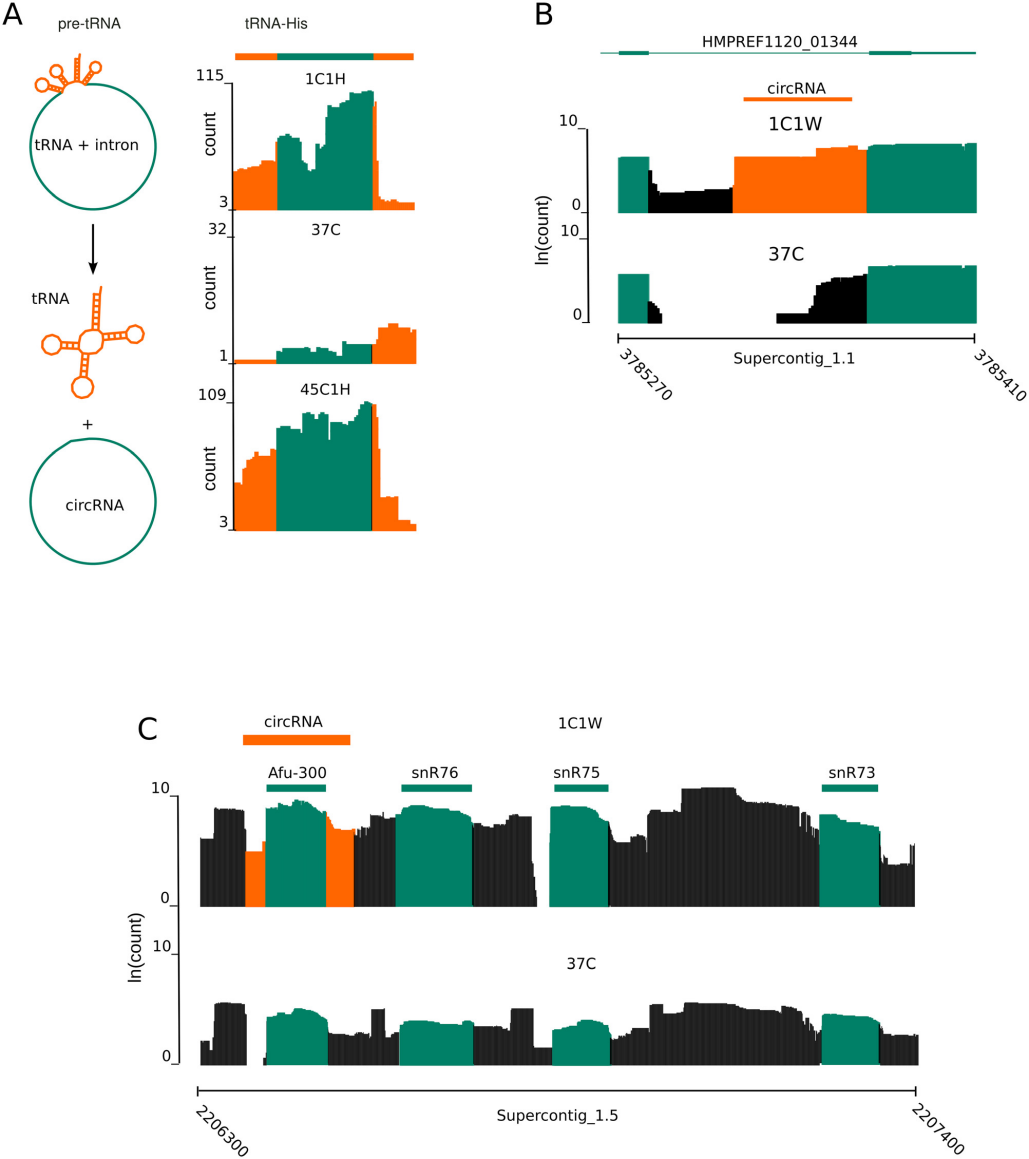
Circular RNAs are differentially expressed. (A) Circular RNA found inside the intron of a tRNA. The two tRNAhalves are shown in orange, while the circular RNA is shown in green. The circularization of the tRNA-His intron happens only after the short-term treatment (1 hour) at 1°C and 45°C. Both halves of the tRNA-His show unconstrained temperature-dependent regulation. *(B)* Log10Count of the reads mapping in the region around the circRNA (orange) located in the intron of HMPREF1120_01344 (green). The expression level of the circRNA is higher upon exposure at 1°C for 1 week than at the control condition (37°C). (C) Expression profile log10(count) at 1°C for 1 week and 37°C of a circRNA overlapping with an homolog of Afu_300 snoRNA (green). For completeness, the snoRNAs belonging to the same snoRNAs cluster are also shown.

All tRNAs, with the exception of trp-tRNAs, were found. 26 tRNAs contain an intron of varying length. Both tRNA-halves and introns of these spliced-tRNAs showed temperature-dependent expression modulation (Fig 4A).

### Circular RNA

Circular RNAs (circRNAs) were investigated by examining reads that contained apparent splice junctions connecting the end (start) of a split read fragment to the start (end) of a downstream (upstream) fragment. The number of such split-reads was strongly influenced by the experimental conditions (S18 Table). The highest number of circular split-reads were found at 1C1H (35877) and the smallest number at 1C1W (4215). Circular RNAs were divided into short circular RNAs, e.g. transcripts covered by circular split reads whose ends were not separated by more than 200 nts and long circular RNAs, where the circular split reads connected distinct exons/introns.

Like the other classes of RNAs, circRNAs show a temperature dependent expression. This is clearly seen for the circRNA located inside HMPREF1120_01344. Its expression is at its peak at 1C1W, while under other conditions, the expression level is strongly down-regulated (Fig 4B). Two cases of circular RNAs overlapping ncRNAs were found. At 1C1H and 45C1H, the intron of the spliced His-tRNA located at Supercontig_1.5 1803599 1803694, is reported to be circularized (21 split reads at 1C1H, 19 split reads at 45C1H) (Fig 4A). Circularized tRNA introns were previously reported in *Haloferax volcanii* [60] and various other archea [61].

The other example is found for 1C1W where the snoRNA homolog to C/D-snoRNA Afu_300 is reported to be located inside a circularized region of 165 nucleotides (Fig 4C). Circularized snoRNAs were previously reported in *Nanoarchaeum equitans [62]* as well as *Pyrococcus furiosus* [61 *]*. The region located around Afu_300 is interesting as it contains a cluster of 4 snoRNAs (snR73, snR76, snR78, Afu_300) that is found in other fungi [63] (Fig 4C).

The circular RNA located in the his-tRNA and the most highly expressed circular RNA at 1C1W (115 sustaining split reads) were tested with RT-PCR (reverse transcriptase PCR) and both were confirmed (see S4 Fig).

### Discussion

In this study we focused on the transcriptome of *E*. *dermatitidis* and its variations under three temperature conditions corresponding to its known environments, i.e. cold water (1°C), human body (37°C) and the warm sites found in different human habitats (45°C).

At 1°C, the cell is mainly focused on becoming adapted to the cold and regulates different processes. The lipid metabolism is the pathway that is most strongly regulated. The modification of the lipidome is a typical response to temperature stresses in both uni- and multicellular organisms [47]. This can be explained by the fact that, at low temperatures, the cell membrane becomes rigid, compromising membrane-associated cellular functions, hindering membrane-bound enzymes, slowing down diffusion rates and inducing cluster formation of integral membrane proteins [64]. However, cells have a large repertoire of processes to increase the membrane fluidity: e.g. the modification of the ratio of polyunsaturated to saturated fatty acids as well as the integration of more sterols into the membrane phosphate bi-layer ensuring membrane integrity and stability [47,53,65]. In our data we found that HMPREF1120_00310, a gene whose yeast homolog is involved in the desaturation of fatty acid, is up-regulated at low temperature and that ergosterol biosynthesis pathway is enriched in genes that are up-regulated at low temperature (p-value 0.02).

Glycerol 3-phosphate metabolism (G3P) is the main active process when the fungus is exposed long-term to 1°C. It has already been established that G3P is mainly involved in the anabolism of triacylglycerols, being the backbone for the biosynthesis of all phospholipids, and it can enter the glycolytic pathway after being oxidized to dihydroxyacetone phosphate. Nevertheless, it is also a major donor of electrons in the mitochondrial respiratory chain. According to our set of genes up-regulated at 1°C, it seems that the latter could be its role under long-term cold exposure (Fig 2D). Thus, the fungus is metabolically active at 1°C.

The heterocyclic catabolism is, after the G3P metabolism, the second most represented process at 1W1C. It leads to the proline catabolic processes and to glutamate biosynthesis (S3 Table). The important role of glutamate in cold stress resistance has previously been reported in different eukaryotes [66–68]. Moreover, Takagi *et al*. have demonstrated how, in yeasts, proline and charged amino acid such as arginine and glutamate exhibit cryoprotective activities almost comparable to that of threalose and glycerol [56]. In summary, with the support of the data related to trehalose pathway, we can conclude that the cryoprotection in this fungus is achieved by the increase of both proline and intracellular trehalose.

Genes related to cell cycle checkpoints are up-regulated after short-term exposure to 1°C, presenting a cell in an alert state. The cell cycle slowdown is a typical response to cold stress, since it has been observed both in prokaryotes [65] and eukaryotes [47,66]. In yeasts, in particular, it is described as a characteristic response to near-freezing temperature (<10°C), although *S*. *cerevisiae* still actively grows when exposed to temperatures between 10 and 18°C [47].

Finally, genes up-regulated over both long and short exposures to 1°C are enriched in GO terms related to the post-chaperonin tubulin folding pathway as well as the tubulin complex biogenesis processes. Interestingly, it was previously reported in *S*. *cerevisiae* that the cytoplasmic chaperonin CCT is a cold shock protein and that its main cellular substrates are actine and tubulin [69].

The number of processes modified by the exposure of *E*. *dermatitidis* at 1°C is much larger than those found at 45°C. For example, no alteration of the lipid-related pathways could be observed at 45°C, indicating that this temperature does not represent a stress condition for the membrane. Furthermore DNA packing and mitotic chromosome condensation are down-regulated during the short 45°C exposure, indicating that the fungus is in a fully replicative state. Taken together the replicative state and the missing modification of membranes indicates that 45°C—albeit being beyond the optimal growth temperature—does not yet induce any cellular stress.

Finally, increased activity of the Golgi apparatus was observed following long-term exposure to 45°C. The importance of the Golgi apparatus at 45C1W is underlined by the enrichment in genes related to the COPII vesicle coat, a type of coat protein that transports other proteins from the endoplasmic reticulum to the Golgi apparatus [70].

Beyond the mere mRNAs, ncRNAs, fusion transcripts as well as circRNAs exhibit temperature-dependent regulation. Moreover, intronic snoRNAs and circRNAs show a regulation pattern different from that of the host-gene. Environmental changes can modulate the expression of house-keeping ncRNAs, like snoRNAs, snRNAs or Rnase [59,71]. With the exception of a few examples [59], the role of these basal RNAs in case of stress conditions is still poorly understood. Even if the function of circRNAs in fungi is still unknown [72,73], their proven resistance to denaturation and to enzymatic degradation [74] might play a role in fungal stress resistance as well as virulence.

## Conclusion

*Exophiala dermatitidis* is an important model organism in view of fungal ecology but also in view of new and emerging mycosis threatening human health. As mentioned in the introduction, the fungus has been isolated from glaciers, from cold and hot tap water, from dishwashers and sauna facilities, and the clinical occurrence of the fungus as an agent of cutaneous, subcutaneous and systemic mycosis is increasing. Hitherto, effective therapies and drugs have not been developed, a consequence of the lack of knowledge of the ecology of the fungus and its virulence factors. In this study the acclimatization of *E*. *dermatitidis* to temperatures ranging from 1°C to 45°C was studied because temperature plays an important role in the fungus pathogenicity. Our molecular observations confirm that *E*. *dermatitidis* can survive at a wide range of temperatures. While it shows some stress responses when exposed to 1°C—like DNA and cell cycle checkpoints and modification of membranes—45°C does not seem to induce any stress response in the cell. However, the fungus—after having acclimatized to 1°C—is metabolically active even at this temperature.

The wide ecological amplitude and the ability to be active at low and high temperatures, which might in part be due to the capacity of the fungus to fuse distinct mRNAs to create the necessary proteins on-the-fly, and is probably responsible for the success of *Exophiala dermatitidis* in thriving from its natural habitat into the warm human environment and as a pathogen into the human host.

Our study is part of an emerging research trend that applies the use of Next Generation Sequencing tools to the study of many relevant pathogenic fungi [75–77] with the aim of deepening the knowledge of the gene expressions that underlie their pathogenicity. Together with the work published in [27] and by Robertson *et al*. [6] that also focus on the transcriptome of *E*. *dermatitidis*, our work is pioneering for this fungus.

Getting a deeper understanding of the cellular mechanisms of the fungus, both on the protein and RNA levels, is a first step towards the understanding of *E*. *dermatitidis* pathogenicity and will pave the way for the development of novel drug targets for effective therapies against the emerging mycosis caused by this fungus.

## Supporting Information

S1 Fig Starch and sucrose metabolic metabolic pathway. Each rectangle correspond to an enzyme. White rectangles correspond to enzymes not found in S. cerevisiae. Green rectangles correspond to enzymes present in S. cerevisiae. Red coloured rectangles are downregulated genes at 1C1W with respect to 37C. Blue coloured rectangles are upregulated genes at 1C1W compared to 37C. ⇡and ⇣represent a up- and downregulation smaller than a factor two. ↑ and ↓ represent a 2 to 4 fold up- and downregulation, respectively. ⇑and ⇓represent a 4 to 8 fold up- and downregulation, respectively. ⤊ and ⤋represent a more than 8 fold up- and downregulation, respectively.
(TIFF)

S2 Fig Expression profile of an intronic snoRNA homolog to Afu_191. The snoRNA corresponds to the green line located in the host gene HMPREF1120_04106 (yellow line). The log10(count) are reported for each temperature. Upon exposure at 1°C for 1 week, the snoRNA is at its highest expression, while the host gene is lowly expressed, clearly indicating that the snoRNA and mRNA expressions are not always related.
(TIFF)

S3 Fig Expression profile of the 10 closest genes with respect to the medoid of cluster 1. (TIFF)

S4 Fig Agarose gel of RT-PCR of tested circular RNAs. cRNA1.4 is the most highly expressed circRNA at 1C1W with 115 sustaining reads. The expected size of the amplicon is 80bp. cRNA1.5a/b are located inside the his-tRNA intron (See Fig 4a). Two different primer pairs were used for cRNA1.5a and cRNA1.5b, leading to an amplicon size of 71bp and 70bp, respectively. The second amplicon at around 700bp is probably due to an unspecific binding of the primers couple on transcript HMPREF1120_08504T0 as reported by RNAplex.
(EPS)

S1 Table List of over-represented GO terms in the Biological Process category for the genes up-regulated at 1°C for 1 hour. (DOC)

S2 Table List of over-represented GO terms in the Biological Process category for the genes down-regulated at 1°C for 1 hour. (DOCX)

S3 Table List of over-represented GO terms in the Biological Process category for the genes up-regulated at 1°C for 1 week. (DOCX)

S4 Table List of over-represented GO terms in the Biological Process category for the genes down-regulated at 1°C for 1 week. (DOCX)

S5 Table List of over-represented GO terms in the Biological Process category for the genes up-regulated at 45°C for 1 hour. (DOCX)

S6 Table List of over-represented GO terms in the Biological Process category for the genes down-regulated at 45°C for 1 hour. (DOCX)

S7 Table List of over-represented GO terms in the Biological Process category for the genes up-regulated at 45°C for 1 week. (DOCX)

S8 Table List of over-represented GO terms in the Biological Process category for the genes down-regulated at 45°C for 1 week. (DOCX)

S9 Table List of over-represented GO terms in the Cellular Components category for the genes down-regulated at 1°C 1 hour. (DOCX)

S10 Table List of over-represented GO terms in the Cellular Components category for the genes up-regulated at 1°C for 1 hour. (DOCX)

S11 Table List of over-represented GO terms in the Cellular Components category for the genes down-regulated at 1°C for 1 week. (DOCX)

S12 Table List of over-represented GO terms in the Cellular Components category for the genes up-regulated at 1°C for 1 week. (DOCX)

S13 Table List of over-represented GO terms in the Cellular Components category for the genes down-regulated at 45°C for 1hour. (DOCX)

S14 Table List of over-represented GO terms in the Cellular Components category for the genes up-regulated at 45°C for 1 hour. (DOCX)

S15 Table List of over-represented GO terms in the Cellular Components category for the genes down-regulated at 45°C for 1 week. (DOCX)

S16 Table List of over-represented GO terms in the Cellular Components category for the genes up-regulated at 45°C for 1 week. (DOCX)

S17 Table Melanin biosynthesis pathway genes regulated by at least a factor 8 compared to the control at 37°C. (DOCX)

S18 Table Number of circular split reads returned by the segemehl:testrealign approach. Splits with the passed flags were retained. * The number of splitreads is at least 10. The distance between the mapping position of the read end and read start is less than 200 nts. **Reads end and Reads start are not on the same exon, intron, the number of splitreads is at least 10 and reads end and start are falling close (less than 20nts) to an intron/exon boundary.
(DOCX)

S19 Table Number of linear splits returned by the segemehl:testrealign approach. Only splits with the passed flags were retained. **The number of split-reads is at least 10 and reads start and end are falling close (less than 20nts) to an intron/exon boundary.
(DOCX)

S20 Table Number of annotated ncRNAs genes for different ncRNA families. (DOCX)

S21 Table Raw number of reads and mapped number of reads for the 5 experimental conditions. (DOCX)
