# Correction: From Glacier to Sauna: RNA-Seq of the Human Pathogen Black Fungus *Exophiala dermatitidis* under Varying Temperature Conditions Exhibits Common and Novel Fungal Response

**DOI:** 10.1371/journal.pone.0208360

**Published:** 2018-11-26

**Authors:** The PLOS ONE Staff

In the Data Availability section, the web address for the data available at GEO is listed incorrectly. The correct address is: https://www.ncbi.nlm.nih.gov/geo/query/acc.cgi?acc=GSE63785. The accession number for this data is: GSE63785. The publisher apologizes for the error.
