# Supplementary figures and images for: From Glacier to Sauna: RNA-Seq of the Human Pathogen Black Fungus Exophiala dermatitidis under Varying Temperature Conditions Exhibits Common and Novel Fungal Response
Source: PLoS One. 2015 Jun 10;10(6):e0127103. doi: 10.1371/journal.pone.0127103 (PMC4463862; doi:10.1371/journal.pone.0127103)

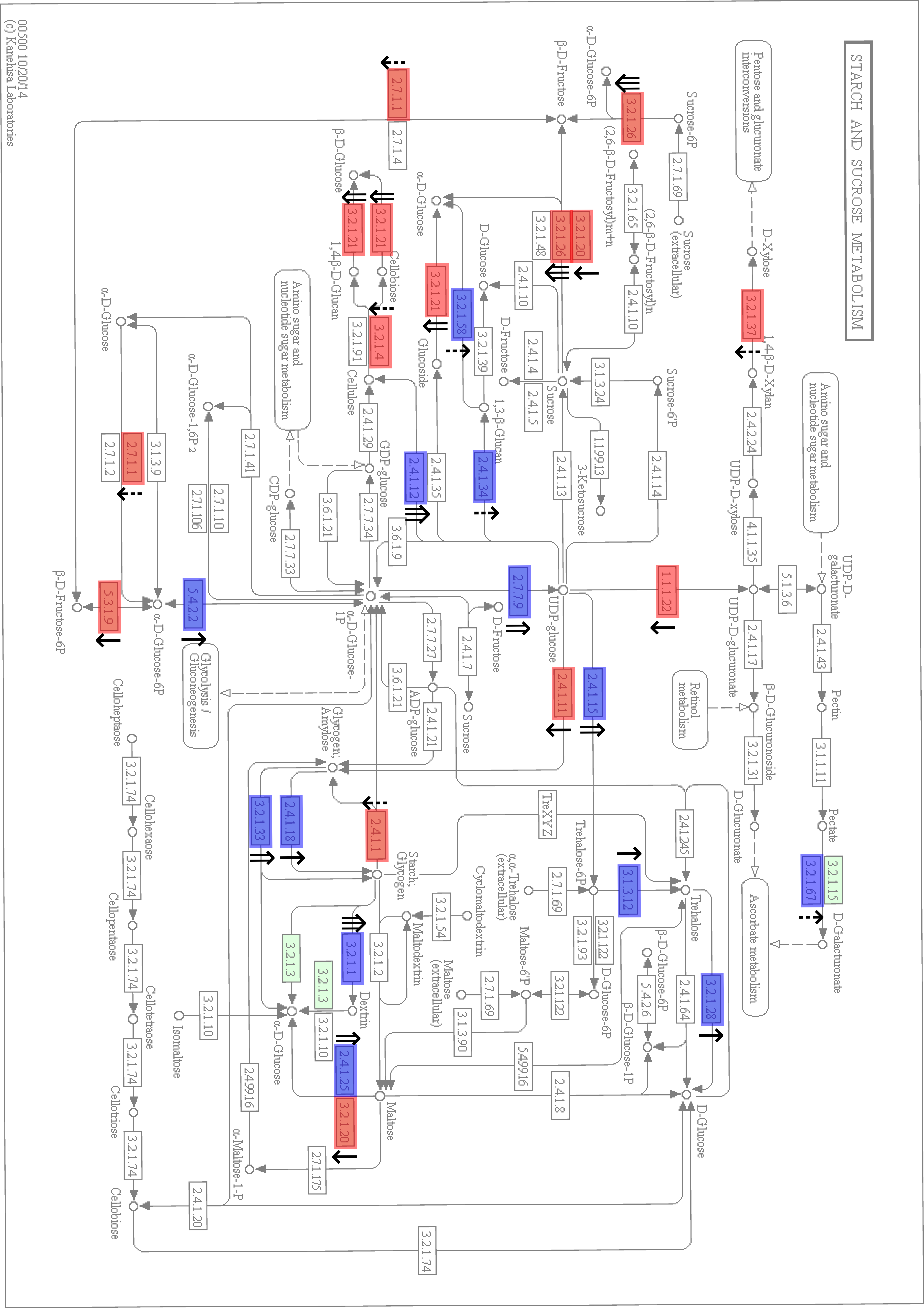

Supplement: S1 Fig — Each rectangle correspond to an enzyme. White rectangles correspond to enzymes not found in S. cerevisiae. Green rectangles correspond to enzymes present in S. cerevisiae. Red coloured rectangles are downregulated genes at 1C1W with respect to 37C. Blue coloured rectangles are upregulated genes at 1C1W compared to 37C. ⇡and ⇣represent a up- and downregulation smaller than a factor two. ↑ and ↓ represent a 2 to 4 fold up- and downregulation, respectively. ⇑and ⇓represent a 4 to 8 fold up- and downregulation, respectively. ⤊ and ⤋represent a more than 8 fold up- and downregulation, respectively. (TIFF) [file pone.0127103.s001.tiff]

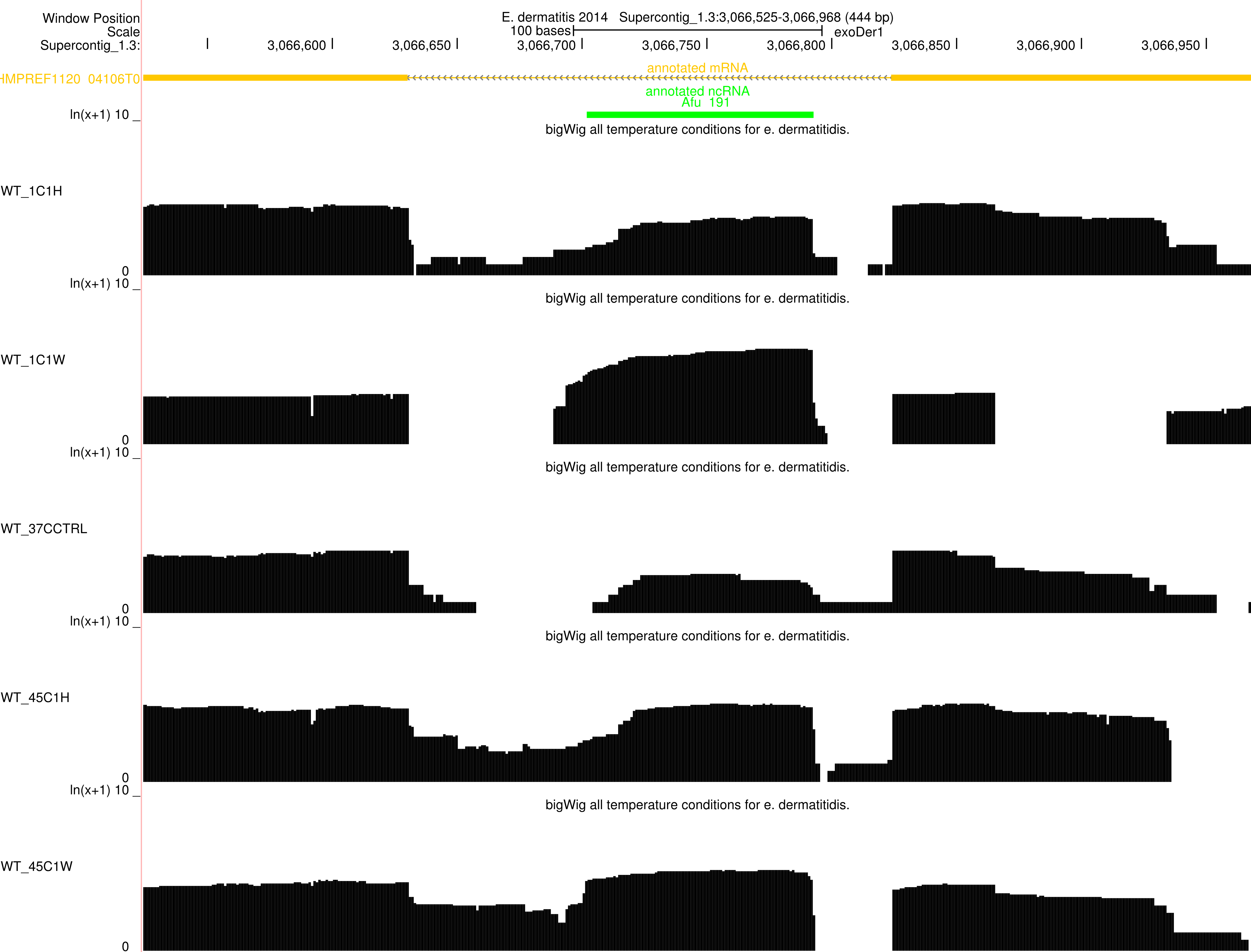

Supplement: S2 Fig — The snoRNA corresponds to the green line located in the host gene HMPREF1120_04106 (yellow line). The log10(count) are reported for each temperature. Upon exposure at 1°C for 1 week, the snoRNA is at its highest expression, while the host gene is lowly expressed, clearly indicating that the snoRNA and mRNA expressions are not always related. (TIFF) [file pone.0127103.s002.tiff]

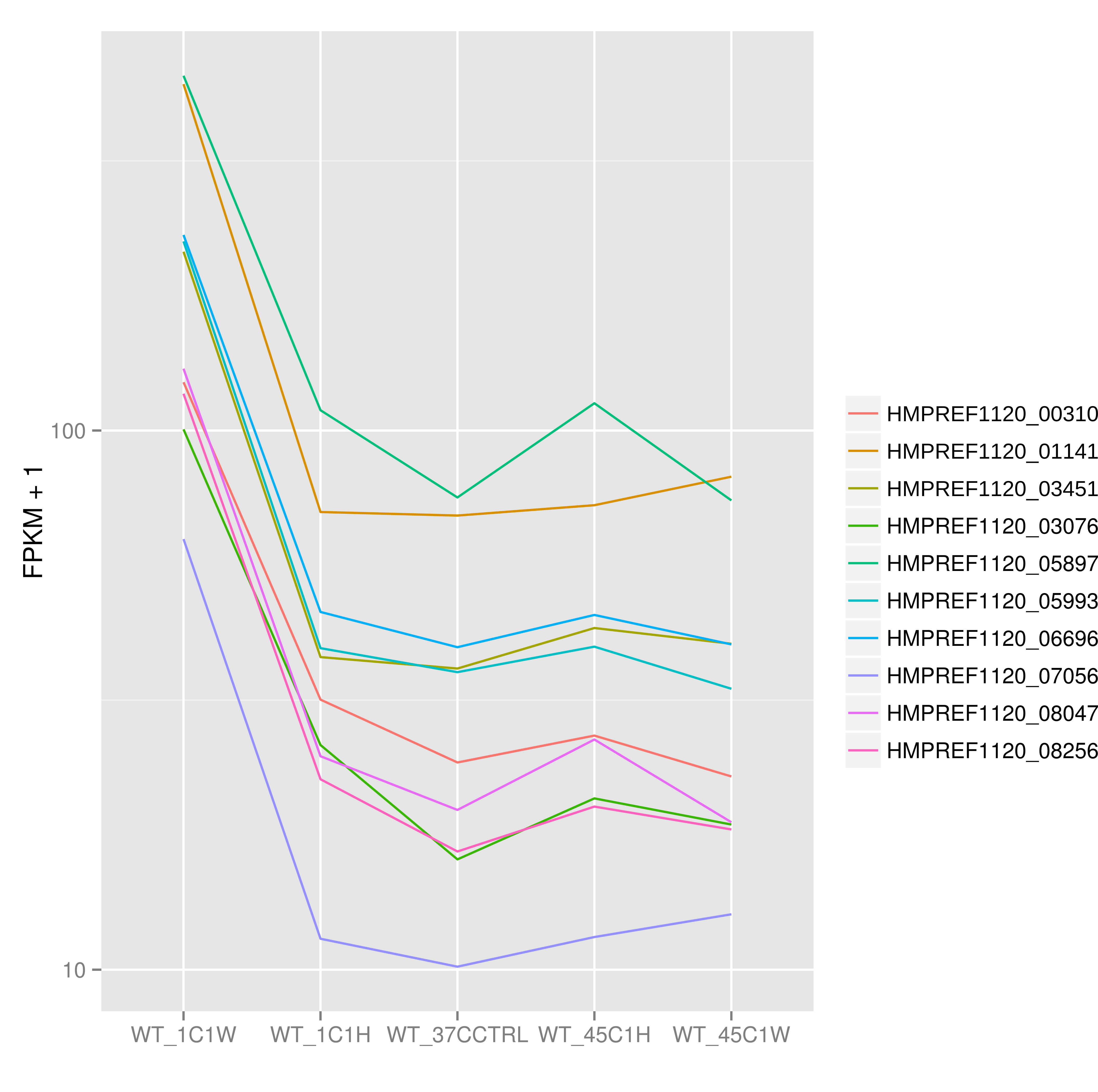

Supplement: S3 Fig — (TIFF) [file pone.0127103.s003.tiff]
