# Supplementary material for: From Glacier to Sauna: RNA-Seq of the Human Pathogen Black Fungus Exophiala dermatitidis under Varying Temperature Conditions Exhibits Common and Novel Fungal Response
Source: PLoS One. 2015 Jun 10;10(6):e0127103. doi: 10.1371/journal.pone.0127103 (PMC4463862; doi:10.1371/journal.pone.0127103)
Supplement: S1 Table — (DOC) [file pone.0127103.s005.doc]

| GO | P-Value | Description |
| --- | --- | --- |
| GO:0006629 | 1.05E-003 | lipid metabolic process |
| GO:0030258 | 6.66E-003 | lipid modification |
| GO:0044238 | 6.81E-003 | primary metabolic process |
| GO:0007023 | 9.05E-003 | post-chaperonin tubulin folding pathway |
| GO:0071704 | 1.76E-002 | organic substance metabolic process |
| GO:0046488 | 1.76E-002 | phosphatidylinositol metabolic process |
| GO:0006635 | 1.80E-002 | fatty acid beta-oxidation |
| GO:0048869 | 1.80E-002 | cellular developmental process |
| GO:0048856 | 1.80E-002 | anatomical structure development |
| GO:0031570 | 1.80E-002 | DNA integrity checkpoint |
| GO:0000902 | 1.80E-002 | cell morphogenesis |
| GO:0019395 | 1.80E-002 | fatty acid oxidation |
| GO:0000077 | 1.80E-002 | DNA damage checkpoint |
| GO:0009653 | 1.80E-002 | anatomical structure morphogenesis |
| GO:0072668 | 1.80E-002 | tubulin complex biogenesis |
| GO:0032989 | 1.80E-002 | cellular component morphogenesis |
| GO:0034440 | 1.80E-002 | lipid oxidation |
| GO:0006650 | 2.23E-002 | glycerophospholipid metabolic process |
| GO:0046486 | 2.23E-002 | glycerolipid metabolic process |
| GO:0032502 | 2.69E-002 | developmental process |
| GO:0044767 | 2.69E-002 | single-organism developmental process |
| GO:0046856 | 3.57E-002 | phosphatidylinositol dephosphorylation |
| GO:0046839 | 3.57E-002 | phospholipid dephosphorylation |
| GO:0044255 | 4.17E-002 | cellular lipid metabolic process |
| GO:0009062 | 4.45E-002 | fatty acid catabolic process |
| GO:0000075 | 4.45E-002 | cell cycle checkpoint |

Supplementary Table 1: List of overrepresented GO terms in the Biological Process category for the genes upregulated at 1C1H
