# Supplementary material for: From Glacier to Sauna: RNA-Seq of the Human Pathogen Black Fungus Exophiala dermatitidis under Varying Temperature Conditions Exhibits Common and Novel Fungal Response
Source: PLoS One. 2015 Jun 10;10(6):e0127103. doi: 10.1371/journal.pone.0127103 (PMC4463862; doi:10.1371/journal.pone.0127103)
Supplement: S2 Table — (DOCX) [file pone.0127103.s006.docx]

| GO | P-Value | Description |
| --- | --- | --- |
| GO:0071103 | 4.24E-003 | DNA conformation change |
| GO:0043174 | 1.89E-002 | nucleoside salvage |
| GO:0006611 | 1.89E-002 | protein export from nucleus |
| GO:0006166 | 1.89E-002 | purine ribonucleoside salvage |
| GO:0006997 | 1.89E-002 | nucleus organization |
| GO:0006998 | 1.89E-002 | nuclear envelope organization |
| GO:0046160 | 1.89E-002 | heme a metabolic process |
| GO:0006784 | 1.89E-002 | heme a biosynthetic process |
| GO:0006259 | 2.21E-002 | DNA metabolic process |
| GO:0007018 | 2.39E-002 | microtubule-based movement |
| GO:0007017 | 2.52E-002 | microtubule-based process |
| GO:0006323 | 3.14E-002 | DNA packaging |
| GO:0007059 | 3.14E-002 | chromosome segregation |
| GO:0006928 | 3.55E-002 | cellular component movement |
| GO:0043101 | 3.75E-002 | purine-containing compound salvage |
| GO:0006405 | 3.75E-002 | RNA export from nucleus |
| GO:0006406 | 3.75E-002 | mRNA export from nucleus |

Supplementary Table 2: List of overrepresented GO terms in the Biological Process category for the genes downregulated at 1C1H
