# Supplementary material for: From Glacier to Sauna: RNA-Seq of the Human Pathogen Black Fungus Exophiala dermatitidis under Varying Temperature Conditions Exhibits Common and Novel Fungal Response
Source: PLoS One. 2015 Jun 10;10(6):e0127103. doi: 10.1371/journal.pone.0127103 (PMC4463862; doi:10.1371/journal.pone.0127103)
Supplement: S3 Table — (DOCX) [file pone.0127103.s007.docx]

| GO | P-Value | Description |
| --- | --- | --- |
| "GO:0046700" | 6.05E-005 | "heterocycle catabolic process" |
| "GO:1901361" | 8.32E-005 | "organic cyclic compound catabolic process" |
| "GO:0044270" | 1.09E-003 | "cellular nitrogen compound catabolic process" |
| "GO:0019439" | 1.09E-003 | "aromatic compound catabolic process" |
| "GO:0072668" | 6.33E-003 | "tubulin complex biogenesis" |
| "GO:0052646" | 8.35E-003 | "alditol phosphate metabolic process" |
| "GO:0006072" | 8.35E-003 | "glycerol-3-phosphate metabolic process" |
| "GO:0006793" | 1.11E-002 | "phosphorus metabolic process" |
| "GO:0019637" | 1.56E-002 | "organophosphate metabolic process" |
| "GO:0006796" | 1.62E-002 | "phosphate-containing compound metabolic process" |
| "GO:0006122" | 1.80E-002 | "mitochondrial electron transport, ubiquinol to cytochrome c" |
| "GO:0006562" | 1.80E-002 | "proline catabolic process" |
| "GO:1901615" | 1.81E-002 | "organic hydroxy compound metabolic process" |
| "GO:1901565" | 2.42E-002 | "organonitrogen compound catabolic process" |
| "GO:0006537" | 3.41E-002 | "glutamate biosynthetic process" |
| "GO:0042775" | 3.41E-002 | "mitochondrial ATP synthesis coupled electron transport" |
| "GO:0018958" | 3.41E-002 | "phenol-containing compound metabolic process" |
| "GO:0019748" | 3.41E-002 | "secondary metabolic process" |
| "GO:1901135" | 3.66E-002 | "carbohydrate derivative metabolic process" |
| "GO:0055086" | 3.66E-002 | "nucleobase-containing small molecule metabolic process" |
| "GO:0019318" | 4.15E-002 | "hexose metabolic process" |
| "GO:0005975" | 4.55E-002 | "carbohydrate metabolic process" |
| "GO:0090407" | 4.77E-002 | "organophosphate biosynthetic process" |
| "GO:0006753" | 4.77E-002 | "nucleoside phosphate metabolic process" |
| "GO:0034655" | 4.95E-002 | "nucleobase-containing compound catabolic process" |

Supplementary Table 3: List of overrepresented GO terms in the Biological Process category for the genes upregulated at 1C1W
