# Supplementary material for: From Glacier to Sauna: RNA-Seq of the Human Pathogen Black Fungus Exophiala dermatitidis under Varying Temperature Conditions Exhibits Common and Novel Fungal Response
Source: PLoS One. 2015 Jun 10;10(6):e0127103. doi: 10.1371/journal.pone.0127103 (PMC4463862; doi:10.1371/journal.pone.0127103)
Supplement: S4 Table — (DOCX) [file pone.0127103.s008.docx]

| GO | P-Value | Description |
| --- | --- | --- |
| "GO:0042254" | 2.70E-005 | "ribosome biogenesis" |
| "GO:0022613" | 4.47E-004 | "ribonucleoprotein complex biogenesis" |
| "GO:0042255" | 3.54E-003 | "ribosome assembly" |
| "GO:0006265" | 3.54E-003 | "DNA topological change" |
| "GO:0006000" | 3.54E-003 | "fructose metabolic process" |
| "GO:0006003" | 3.54E-003 | "fructose 2,6-bisphosphate metabolic process" |
| "GO:0044085" | 1.10E-002 | "cellular component biogenesis" |
| "GO:0006904" | 1.65E-002 | "vesicle docking involved in exocytosis" |
| "GO:0070925" | 1.65E-002 | "organelle assembly" |
| "GO:0006364" | 1.97E-002 | "rRNA processing" |
| "GO:0016072" | 2.30E-002 | "rRNA metabolic process" |
| "GO:0051649" | 2.44E-002 | "establishment of localization in cell" |
| "GO:0022406" | 2.95E-002 | "membrane docking" |
| "GO:0006913" | 2.95E-002 | "nucleocytoplasmic transport" |
| "GO:0051169" | 2.95E-002 | "nuclear transport" |
| "GO:0048278" | 2.95E-002 | "vesicle docking" |
| "GO:0016192" | 2.95E-002 | "vesicle-mediated transport" |
| "GO:0043174" | 3.50E-002 | "nucleoside salvage" |
| "GO:0043967" | 3.50E-002 | "histone H4 acetylation" |
| "GO:0043968" | 3.50E-002 | "histone H2A acetylation" |
| "GO:0042256" | 3.50E-002 | "mature ribosome assembly" |
| "GO:0006611" | 3.50E-002 | "protein export from nucleus" |
| "GO:0006475" | 3.50E-002 | "internal protein amino acid acetylation" |
| "GO:0032501" | 3.50E-002 | "multicellular organismal process" |
| "GO:0044707" | 3.50E-002 | "single-multicellular organism process" |
| "GO:0006166" | 3.50E-002 | "purine ribonucleoside salvage" |
| "GO:0006997" | 3.50E-002 | "nucleus organization" |
| "GO:0006998" | 3.50E-002 | "nuclear envelope organization" |
| "GO:0016573" | 3.50E-002 | "histone acetylation" |
| "GO:0046168" | 3.50E-002 | "glycerol-3-phosphate catabolic process" |
| "GO:0007275" | 3.50E-002 | "multicellular organismal development" |
| "GO:0031122" | 3.50E-002 | "cytoplasmic microtubule organization" |
| "GO:0007050" | 3.50E-002 | "cell cycle arrest" |
| "GO:0018393" | 3.50E-002 | "internal peptidyl-lysine acetylation" |
| "GO:0018394" | 3.50E-002 | "peptidyl-lysine acetylation" |
| "GO:0046040" | 3.70E-002 | "IMP metabolic process" |
| "GO:0006188" | 3.70E-002 | "IMP biosynthetic process" |
| "GO:0006887" | 4.53E-002 | "exocytosis" |

Supplementary Table 4: List of overrepresented GO terms in the Biological Process category for the genes downregulated at 1C1W
