# Supplementary material for: From Glacier to Sauna: RNA-Seq of the Human Pathogen Black Fungus Exophiala dermatitidis under Varying Temperature Conditions Exhibits Common and Novel Fungal Response
Source: PLoS One. 2015 Jun 10;10(6):e0127103. doi: 10.1371/journal.pone.0127103 (PMC4463862; doi:10.1371/journal.pone.0127103)
Supplement: S5 Table — (DOCX) [file pone.0127103.s009.docx]

| GO | P-Value | Description |
| --- | --- | --- |
| "GO:0009262" | 1.37E-004 | "deoxyribonucleotide metabolic process" |
| "GO:0009219" | 1.37E-004 | "pyrimidine deoxyribonucleotide metabolic process" |
| "GO:0019692" | 1.37E-004 | "deoxyribose phosphate metabolic process" |
| "GO:0009394" | 1.37E-004 | "2'-deoxyribonucleotide metabolic process" |
| "GO:0006220" | 2.00E-003 | "pyrimidine nucleotide metabolic process" |
| "GO:0072527" | 5.21E-003 | "pyrimidine-containing compound metabolic process" |
| "GO:0046080" | 6.99E-003 | "dUTP metabolic process" |
| "GO:0046072" | 6.99E-003 | "dTDP metabolic process" |
| "GO:0009196" | 6.99E-003 | "pyrimidine deoxyribonucleoside diphosphate metabolic process" |
| "GO:0009197" | 6.99E-003 | "pyrimidine deoxyribonucleoside diphosphate biosynthetic process" |
| "GO:0009189" | 6.99E-003 | "deoxyribonucleoside diphosphate biosynthetic process" |
| "GO:0009138" | 6.99E-003 | "pyrimidine nucleoside diphosphate metabolic process" |
| "GO:0009139" | 6.99E-003 | "pyrimidine nucleoside diphosphate biosynthetic process" |
| "GO:0006233" | 6.99E-003 | "dTDP biosynthetic process" |
| "GO:0009186" | 1.39E-002 | "deoxyribonucleoside diphosphate metabolic process" |
| "GO:0009133" | 1.39E-002 | "nucleoside diphosphate biosynthetic process" |
| "GO:0009263" | 1.39E-002 | "deoxyribonucleotide biosynthetic process" |
| "GO:0009265" | 1.39E-002 | "2'-deoxyribonucleotide biosynthetic process" |
| "GO:0009221" | 1.39E-002 | "pyrimidine deoxyribonucleotide biosynthetic process" |
| "GO:0009211" | 1.39E-002 | "pyrimidine deoxyribonucleoside triphosphate metabolic process" |
| "GO:0006419" | 1.39E-002 | "alanyl-tRNA aminoacylation" |
| "GO:0046385" | 1.39E-002 | "deoxyribose phosphate biosynthetic process" |
| "GO:0009132" | 2.08E-002 | "nucleoside diphosphate metabolic process" |
| "GO:0009200" | 2.08E-002 | "deoxyribonucleoside triphosphate metabolic process" |
| "GO:0046856" | 2.77E-002 | "phosphatidylinositol dephosphorylation" |
| "GO:0046839" | 2.77E-002 | "phospholipid dephosphorylation" |
| "GO:0009147" | 3.45E-002 | "pyrimidine nucleoside triphosphate metabolic process" |
| "GO:0006270" | 4.80E-002 | "DNA replication initiation" |

Supplementary Table 5: List of overrepresented GO terms in the Biological Process category for the genes upregulated at 45C1H
