# Supplementary material for: From Glacier to Sauna: RNA-Seq of the Human Pathogen Black Fungus Exophiala dermatitidis under Varying Temperature Conditions Exhibits Common and Novel Fungal Response
Source: PLoS One. 2015 Jun 10;10(6):e0127103. doi: 10.1371/journal.pone.0127103 (PMC4463862; doi:10.1371/journal.pone.0127103)
Supplement: S6 Table — (DOCX) [file pone.0127103.s010.docx]

| GO | P-Value | Description |
| --- | --- | --- |
| "GO:0071103" | 1.10E-003 | "DNA conformation change" |
| "GO:0043174" | 1.19E-002 | "nucleoside salvage" |
| "GO:0006611" | 1.19E-002 | "protein export from nucleus" |
| "GO:0006166" | 1.19E-002 | "purine ribonucleoside salvage" |
| "GO:0006997" | 1.19E-002 | "nucleus organization" |
| "GO:0006998" | 1.19E-002 | "nuclear envelope organization" |
| "GO:0016598" | 1.19E-002 | "protein arginylation" |
| "GO:0019295" | 1.19E-002 | "coenzyme M biosynthetic process" |
| "GO:0019296" | 1.19E-002 | "coenzyme M metabolic process" |
| "GO:0006323" | 1.31E-002 | "DNA packaging" |
| "GO:0043101" | 2.37E-002 | "purine-containing compound salvage" |
| "GO:0006405" | 2.37E-002 | "RNA export from nucleus" |
| "GO:0006406" | 2.37E-002 | "mRNA export from nucleus" |
| "GO:0017182" | 3.54E-002 | "peptidyl-diphthamide metabolic process" |
| "GO:0017183" | 3.54E-002 | "peptidyl-diphthamide biosynthetic process from peptidyl-histidine" |
| "GO:0018202" | 3.54E-002 | "peptidyl-histidine modification" |
| "GO:0006265" | 3.54E-002 | "DNA topological change" |
| "GO:0051168" | 3.54E-002 | "nuclear export" |
| "GO:0009395" | 3.54E-002 | "phospholipid catabolic process" |
| "GO:0051028" | 4.69E-002 | "mRNA transport" |
| "GO:0035383" | 4.69E-002 | "thioester metabolic process" |
| "GO:0006637" | 4.69E-002 | "acyl-CoA metabolic process" |
| "GO:0030261" | 4.69E-002 | "chromosome condensation" |
| "GO:0006487" | 4.69E-002 | "protein N-linked glycosylation" |
| "GO:0006403" | 4.69E-002 | "RNA localization" |
| "GO:0050657" | 4.69E-002 | "nucleic acid transport" |
| "GO:0050658" | 4.69E-002 | "RNA transport" |
| "GO:0051236" | 4.69E-002 | "establishment of RNA localization" |
| "GO:0007076" | 4.69E-002 | "mitotic chromosome condensation" |
| "GO:0006732" | 4.72E-002 | "coenzyme metabolic process" |

Supplementary Table 6: List of overrepresented GO terms in the Biological Process category for the genes downregulated at 45C1H
