# Supplementary material for: From Glacier to Sauna: RNA-Seq of the Human Pathogen Black Fungus Exophiala dermatitidis under Varying Temperature Conditions Exhibits Common and Novel Fungal Response
Source: PLoS One. 2015 Jun 10;10(6):e0127103. doi: 10.1371/journal.pone.0127103 (PMC4463862; doi:10.1371/journal.pone.0127103)
Supplement: S7 Table — (DOCX) [file pone.0127103.s011.docx]

| GO | P-Value | Description |
| --- | --- | --- |
| "GO:0046854" | 2.61E-002 | "phosphatidylinositol phosphorylation" |
| "GO:0046834" | 2.61E-002 | "lipid phosphorylation" |
| "GO:0043043" | 3.25E-002 | "peptide biosynthetic process" |
| "GO:0019184" | 3.25E-002 | "nonribosomal peptide biosynthetic process" |
| "GO:0006750" | 3.25E-002 | "glutathione biosynthetic process" |
| "GO:0015031" | 4.73E-002 | "protein transport" |

Supplementary Table 7: List of overrepresented GO terms in the Biological Process category for the genes upregulated at 45C1W
