# Supplementary material for: From Glacier to Sauna: RNA-Seq of the Human Pathogen Black Fungus Exophiala dermatitidis under Varying Temperature Conditions Exhibits Common and Novel Fungal Response
Source: PLoS One. 2015 Jun 10;10(6):e0127103. doi: 10.1371/journal.pone.0127103 (PMC4463862; doi:10.1371/journal.pone.0127103)
Supplement: S8 Table — (DOCX) [file pone.0127103.s012.docx]

| GO | P-Value | Description |
| --- | --- | --- |
| "GO:0018130" | 2.50E-003 | "heterocycle biosynthetic process" |
| "GO:1901362" | 3.36E-003 | "organic cyclic compound biosynthetic process" |
| "GO:0046483" | 3.87E-003 | "heterocycle metabolic process" |
| "GO:1901360" | 5.52E-003 | "organic cyclic compound metabolic process" |
| "GO:0046040" | 6.06E-003 | "IMP metabolic process" |
| "GO:0006188" | 6.06E-003 | "IMP biosynthetic process" |
| "GO:0044271" | 7.97E-003 | "cellular nitrogen compound biosynthetic process" |
| "GO:0034641" | 1.17E-002 | "cellular nitrogen compound metabolic process" |
| "GO:0043174" | 1.36E-002 | "nucleoside salvage" |
| "GO:0006611" | 1.36E-002 | "protein export from nucleus" |
| "GO:0000910" | 1.36E-002 | "cytokinesis" |
| "GO:0006166" | 1.36E-002 | "purine ribonucleoside salvage" |
| "GO:0006997" | 1.36E-002 | "nucleus organization" |
| "GO:0006998" | 1.36E-002 | "nuclear envelope organization" |
| "GO:0016598" | 1.36E-002 | "protein arginylation" |
| "GO:0046160" | 1.36E-002 | "heme a metabolic process" |
| "GO:0006784" | 1.36E-002 | "heme a biosynthetic process" |
| "GO:0072522" | 1.39E-002 | "purine-containing compound biosynthetic process" |
| "GO:0006807" | 1.63E-002 | "nitrogen compound metabolic process" |
| "GO:0019438" | 1.65E-002 | "aromatic compound biosynthetic process" |
| "GO:1901566" | 1.84E-002 | "organonitrogen compound biosynthetic process" |
| "GO:0034654" | 1.98E-002 | "nucleobase-containing compound biosynthetic process" |
| "GO:0006139" | 2.21E-002 | "nucleobase-containing compound metabolic process" |
| "GO:0006725" | 2.30E-002 | "cellular aromatic compound metabolic process" |
| "GO:1901137" | 2.33E-002 | "carbohydrate derivative biosynthetic process" |
| "GO:0071103" | 2.39E-002 | "DNA conformation change" |
| "GO:0043101" | 2.70E-002 | "purine-containing compound salvage" |
| "GO:0006405" | 2.70E-002 | "RNA export from nucleus" |
| "GO:0006406" | 2.70E-002 | "mRNA export from nucleus" |
| "GO:0070085" | 3.48E-002 | "glycosylation" |
| "GO:0072521" | 3.87E-002 | "purine-containing compound metabolic process" |
| "GO:0000460" | 4.02E-002 | "maturation of 5.8S rRNA" |
| "GO:0000463" | 4.02E-002 | "maturation of LSU-rRNA from tricistronic rRNA transcript (SSU-rRNA, 5.8S rRNA, LSU-rRNA)" |
| "GO:0000466" | 4.02E-002 | "maturation of 5.8S rRNA from tricistronic rRNA transcript (SSU-rRNA, 5.8S rRNA, LSU-rRNA)" |
| "GO:0000470" | 4.02E-002 | "maturation of LSU-rRNA" |
| "GO:0006265" | 4.02E-002 | "DNA topological change" |
| "GO:0051168" | 4.02E-002 | "nuclear export" |

Supplementary Table 8: List of overrepresented GO terms in the Biological Process category for the genes downregulated at 45C1W
