# Supplementary material for: From Glacier to Sauna: RNA-Seq of the Human Pathogen Black Fungus Exophiala dermatitidis under Varying Temperature Conditions Exhibits Common and Novel Fungal Response
Source: PLoS One. 2015 Jun 10;10(6):e0127103. doi: 10.1371/journal.pone.0127103 (PMC4463862; doi:10.1371/journal.pone.0127103)
Supplement: S9 Table — (DOCX) [file pone.0127103.s013.docx]

| GO | P-Value | Description |
| --- | --- | --- |
| "GO:0032993" | 8.89E-003 | "protein-DNA complex" |
| "GO:0044445" | 1.13E-002ooo | "cytosolic part" |
| "GO:0005635" | 1.13E-002 | "nuclear envelope" |
| "GO:0044815" | 1.34E-002 | "DNA packaging complex" |
| "GO:0005694" | 1.47E-002 | "chromosome" |
| "GO:0005868" | 1.52E-002 | "cytoplasmic dynein complex" |
| "GO:0043234" | 1.53E-002 | "protein complex" |
| "GO:0015630" | 1.74E-002 | "microtubule cytoskeleton" |
| "GO:0005829" | 2.07E-002 | "cytosol" |
| "GO:0044422" | 2.69E-002 | "organelle part" |
| "GO:0044446" | 2.69E-002 | "intracellular organelle part" |
| "GO:0005875" | 2.93E-002 | "microtubule associated complex" |
| "GO:0009316" | 3.01E-002 | "3-isopropylmalate dehydratase complex" |
| "GO:0005835" | 3.01E-002 | "fatty acid synthase complex" |
| "GO:0031965" | 3.01E-002 | "nuclear membrane" |
| "GO:0030286" | 3.01E-002 | "dynein complex" |
| "GO:0044427" | 3.84E-002 | "chromosomal part" |
| "GO:0044428" | 3.88E-002 | "nuclear part" |
| "GO:0000793" | 3.91E-002 | "condensed chromosome" |
| "GO:0000796" | 4.48E-002 | "condensin complex" |
| "GO:0030687" | 4.48E-002 | "preribosome, large subunit precursor" |
| "GO:0044430" | 4.67E-002 | "cytoskeletal part" |

Supplementary Table 9: List of overrepresented GO terms in the Cellular Components category for the genes downregulated at 1C1H
