# Supplementary material for: From Glacier to Sauna: RNA-Seq of the Human Pathogen Black Fungus Exophiala dermatitidis under Varying Temperature Conditions Exhibits Common and Novel Fungal Response
Source: PLoS One. 2015 Jun 10;10(6):e0127103. doi: 10.1371/journal.pone.0127103 (PMC4463862; doi:10.1371/journal.pone.0127103)
Supplement: S10 Table — (DOCX) [file pone.0127103.s014.docx]

| GO | P-Value | Description |
| --- | --- | --- |
| "GO:0030896" | 2.32E-002 | "checkpoint clamp complex" |

Supplementary Table 10: List of overrepresented GO terms in the Cellular Components category for the genes upregulated at 1C1H
