# Supplementary material for: From Glacier to Sauna: RNA-Seq of the Human Pathogen Black Fungus Exophiala dermatitidis under Varying Temperature Conditions Exhibits Common and Novel Fungal Response
Source: PLoS One. 2015 Jun 10;10(6):e0127103. doi: 10.1371/journal.pone.0127103 (PMC4463862; doi:10.1371/journal.pone.0127103)
Supplement: S11 Table — (DOCX) [file pone.0127103.s015.docx]

| GO | P-Value | Description |
| --- | --- | --- |
| "GO:0005635" | 6.84E-003 | "nuclear envelope" |
| "GO:0031314" | 3.77E-002 | "extrinsic component of mitochondrial inner membrane" |
| "GO:0000930" | 3.77E-002 | "gamma-tubulin complex" |
| "GO:0005868" | 3.77E-002 | "cytoplasmic dynein complex" |
| "GO:0044450" | 3.77E-002 | "microtubule organizing center part" |
| "GO:0046930" | 4.25E-002 | "pore complex" |
| "GO:0005643" | 4.25E-002 | "nuclear pore" |

Supplementary Table 11: List of overrepresented GO terms in the Cellular Components category for the genes downregulated at 1C1W
