# Supplementary material for: From Glacier to Sauna: RNA-Seq of the Human Pathogen Black Fungus Exophiala dermatitidis under Varying Temperature Conditions Exhibits Common and Novel Fungal Response
Source: PLoS One. 2015 Jun 10;10(6):e0127103. doi: 10.1371/journal.pone.0127103 (PMC4463862; doi:10.1371/journal.pone.0127103)
Supplement: S12 Table — (DOCX) [file pone.0127103.s016.docx]

| GO | P-Value | Description |
| --- | --- | --- |
| "GO:0070069" | 5.66E-004 | "cytochrome complex" |
| "GO:0005746" | 2.12E-003 | "mitochondrial respiratory chain" |
| "GO:0005750" | 6.88E-003 | "mitochondrial respiratory chain complex III" |
| "GO:0045275" | 6.88E-003 | "respiratory chain complex III" |
| "GO:1990204" | 9.58E-003 | "oxidoreductase complex" |
| "GO:0016021" | 1.02E-002 | "integral component of membrane" |
| "GO:0031224" | 1.02E-002 | "intrinsic component of membrane" |
| "GO:1902495" | 1.95E-002 | "transmembrane transporter complex" |
| "GO:0005874" | 2.08E-002 | "microtubule" |
| "GO:0044425" | 2.45E-002 | "membrane part" |
| "GO:0015630" | 2.96E-002 | "microtubule cytoskeleton" |
| "GO:0044455" | 3.09E-002 | "mitochondrial membrane part" |
| "GO:0009277" | 3.69E-002 | "fungal-type cell wall" |
| "GO:0031300" | 4.66E-002 | "intrinsic component of organelle membrane" |
| "GO:0031301" | 4.66E-002 | "integral component of organelle membrane" |
| "GO:0016020" | 4.81E-002 | "membrane" |

Supplementary Table 12: List of overrepresented GO terms in the Cellular Components category for the genes upregulated at 1C1W
