# Supplementary material for: From Glacier to Sauna: RNA-Seq of the Human Pathogen Black Fungus Exophiala dermatitidis under Varying Temperature Conditions Exhibits Common and Novel Fungal Response
Source: PLoS One. 2015 Jun 10;10(6):e0127103. doi: 10.1371/journal.pone.0127103 (PMC4463862; doi:10.1371/journal.pone.0127103)
Supplement: S13 Table — (DOCX) [file pone.0127103.s017.docx]

| GO | P-Value | Description |
| --- | --- | --- |
| "GO:0044815" | 5.40E-003 | "DNA packaging complex" |
| "GO:0031965" | 1.90E-002 | "nuclear membrane" |
| "GO:0005694" | 2.15E-002 | "chromosome" |
| "GO:0000796" | 2.83E-002 | "condensin complex" |
| "GO:0032993" | 3.01E-002 | "protein-DNA complex" |
| "GO:0005744" | 3.76E-002 | "mitochondrial inner membrane presequence translocase complex" |

Supplementary Table 13: List of overrepresented GO terms in the Cellular Components category for the genes downregulated at 45C1H
