# Supplementary material for: From Glacier to Sauna: RNA-Seq of the Human Pathogen Black Fungus Exophiala dermatitidis under Varying Temperature Conditions Exhibits Common and Novel Fungal Response
Source: PLoS One. 2015 Jun 10;10(6):e0127103. doi: 10.1371/journal.pone.0127103 (PMC4463862; doi:10.1371/journal.pone.0127103)
Supplement: S15 Table — (DOCX) [file pone.0127103.s019.docx]

| GO | P-Value | Description |
| --- | --- | --- |
| "GO:0005635" | 8.34E-003 | "nuclear envelope" |
| "GO:0005868" | 1.30E-002 | "cytoplasmic dynein complex" |
| "GO:0005875" | 2.19E-002 | "microtubule associated complex" |
| "GO:0009316" | 2.58E-002 | "3-isopropylmalate dehydratase complex" |
| "GO:0005869" | 2.58E-002 | "dynactin complex" |
| "GO:0031965" | 2.58E-002 | "nuclear membrane" |
| "GO:0030286" | 2.58E-002 | "dynein complex" |
| "GO:0030687" | 3.85E-002 | "preribosome, large subunit precursor" |
| "GO:0031967" | 4.29E-002 | "organelle envelope" |
| "GO:0031975" | 4.47E-002 | "envelope" |

Supplementary Table 15: List of overrepresented GO terms in the Cellular Components category for the genes downregulated at 45C1W
