# Supplementary material for: From Glacier to Sauna: RNA-Seq of the Human Pathogen Black Fungus Exophiala dermatitidis under Varying Temperature Conditions Exhibits Common and Novel Fungal Response
Source: PLoS One. 2015 Jun 10;10(6):e0127103. doi: 10.1371/journal.pone.0127103 (PMC4463862; doi:10.1371/journal.pone.0127103)
Supplement: S16 Table — (DOCX) [file pone.0127103.s020.docx]

| GO | P-Value | Description |
| --- | --- | --- |
| "GO:0030127" | 2.19E-002 | "COPII vesicle coat" |
| "GO:0030134" | 2.19E-002 | "ER to Golgi transport vesicle" |
| "GO:0012507" | 2.19E-002 | "ER to Golgi transport vesicle membrane" |
| "GO:0030133" | 3.63E-002 | "transport vesicle" |
| "GO:0030658" | 3.63E-002 | "transport vesicle membrane" |

Supplementary Table 16: List of overrepresented GO terms in the Cellular Components category for the genes upregulated at 45C1W
