# Supplementary material for: From Glacier to Sauna: RNA-Seq of the Human Pathogen Black Fungus Exophiala dermatitidis under Varying Temperature Conditions Exhibits Common and Novel Fungal Response
Source: PLoS One. 2015 Jun 10;10(6):e0127103. doi: 10.1371/journal.pone.0127103 (PMC4463862; doi:10.1371/journal.pone.0127103)
Supplement: S17 Table — (DOCX) [file pone.0127103.s021.docx]

| Gene Id | Condition | log2fold | Pathways |
| --- | --- | --- | --- |
| HMPREF1120_00199 | WT_1C1H | 3.62804 | DOPA-melanin |
| HMPREF1120_02312 | WT_1C1W | 4.24166 | DHN-melanin |
| HMPREF1120_02754 | WT_1C1W | -4.68016 | DOPA-melanin |
| HMPREF1120_03345 | WT_1C1W | -3.1387 | DOPA-melanin |
| HMPREF1120_03825 | WT_1C1W | -3.06595 | L-Tyrosine |
| HMPREF1120_04514 | WT_1C1W | -3.50263 | DOPA-melanin |
| HMPREF1120_05316 | WT_1C1W | -3.08518 | DOPA-melanin |
| HMPREF1120_05645 | WT_1C1H | 3.78602 | DHN-melanin |
| HMPREF1120_07724 | WT_1C1W | -4.13352 | DHN-melanin |

Supplementary Table 17: Melanin biosynthesis pathway genes regulated by at least a factor 8 compared to 37C
