# Supplementary material for: From Glacier to Sauna: RNA-Seq of the Human Pathogen Black Fungus Exophiala dermatitidis under Varying Temperature Conditions Exhibits Common and Novel Fungal Response
Source: PLoS One. 2015 Jun 10;10(6):e0127103. doi: 10.1371/journal.pone.0127103 (PMC4463862; doi:10.1371/journal.pone.0127103)
Supplement: S18 Table — Splits with the passed flags were retained. * The number of splitreads is at least 10. The distance between the mapping position of the read end and read start is less than 200 nts. **Reads end and Reads start are not on the same exon, intron, the number of splitreads is at least 10 and reads end and start are falling close (less than 20nts) to an intron/exon boundary. (DOCX) [file pone.0127103.s022.docx]

| Condition | Total Number of Circular Splits | Number of short circular Splits * | Number of circular splits connecting more than 1 exon/intron** |
| --- | --- | --- | --- |
| 1C1H | 35877 | 65 | 240 |
| 1C1W | 4215 | 412 | 667 |
| 37C | 11444 | 23 | 87 |
| 45C1H | 17992 | 151 | 361 |
| 45C1W | 24837 | 96 | 199 |

Supplementary Table 18: Number of circular split reads returned by the segemehl:testrealign approach. Only split with the passed flags were retained. * The number of splitreads is at least 10. The distance between the mapping position of the read end and read start is less than 200 nts. **Reads end and Reads start are not on the same exon, intron, the number of splitreads is at least 10 and reads end and start are falling close (less than 20nts) to an intron/exon boundaries.
