# Supplementary material for: From Glacier to Sauna: RNA-Seq of the Human Pathogen Black Fungus Exophiala dermatitidis under Varying Temperature Conditions Exhibits Common and Novel Fungal Response
Source: PLoS One. 2015 Jun 10;10(6):e0127103. doi: 10.1371/journal.pone.0127103 (PMC4463862; doi:10.1371/journal.pone.0127103)
Supplement: S19 Table — Only splits with the passed flags were retained. **The number of split-reads is at least 10 and reads start and end are falling close (less than 20nts) to an intron/exon boundary. (DOCX) [file pone.0127103.s023.docx]

| Condition | Total Number of linear splits | Number of short linear splits | Number of short strandsplits | Number of long splits | Number of splits connecting more than 1 exon/intron** |
| --- | --- | --- | --- | --- | --- |
| 1C1H | 71777 | 8422 | 252 | 62503 | 218 |
| 1C1W | 277565 | 9622 | 938 | 267005 | 873 |
| 37C | 25619 | 8202 | 64 | 17535 | 82 |
| 45C1H | 124408 | 8841 | 436 | 115131 | 417 |
| 45C1W | 64997 | 9037 | 220 | 55740 | 209 |

Supplementary Table 19: Number of linear splits returned by the segemehl:testrealign approach. Only split with the passed flags were retained. **The number of spliteads is at least 10 and reads start and end are falling close (less than 20nts) to a intron/exon boundaries.
