# Supplementary material for: From Glacier to Sauna: RNA-Seq of the Human Pathogen Black Fungus Exophiala dermatitidis under Varying Temperature Conditions Exhibits Common and Novel Fungal Response
Source: PLoS One. 2015 Jun 10;10(6):e0127103. doi: 10.1371/journal.pone.0127103 (PMC4463862; doi:10.1371/journal.pone.0127103)
Supplement: S20 Table — (DOCX) [file pone.0127103.s024.docx]

| NcRNA family | Count |
| --- | --- |
| snoRNA | 35 |
| tRNA | 142 |
| U1 | 1 |
| U2 | 1 |
| U3 | 1 |
| U4 | 1 |
| U5 | 1 |
| U6 | 1 |
| RNaseP | 1 |
| RNaseMRP | 1 |
| 5S rRNA | 17 |
| 18S rRNA | 1 |

Supplementary Table 20: Number of annotated ncRNAs genes for different ncRNA families.
