# Supplementary material for: From Glacier to Sauna: RNA-Seq of the Human Pathogen Black Fungus Exophiala dermatitidis under Varying Temperature Conditions Exhibits Common and Novel Fungal Response
Source: PLoS One. 2015 Jun 10;10(6):e0127103. doi: 10.1371/journal.pone.0127103 (PMC4463862; doi:10.1371/journal.pone.0127103)
Supplement: S21 Table — (DOCX) [file pone.0127103.s025.docx]

| Experiment | Raw Number of Reads | Mapped Reads |
| --- | --- | --- |
| 1^o^C 1 Hour | 99965344 | 83218193 |
| 1 C 1 Week | 77860005 | 69087910 |
| 37 C | 96207066 | 36992997 |
| 45 C 1 Hour | 57611573 | 40914954 |
| 45 C 1 Week | 69858164 | 41653989 |

Supplementary Table 21: Raw number of reads and mapped number of reads for the 5 experimental conditions
